# Prevalence and influencing factors of abnormal carotid artery intima-media thickness in Henan Province in China

**DOI:** 10.3389/fendo.2023.1266207

**Published:** 2023-10-20

**Authors:** Tingting Yang, Yating Wang, Xiaoke Zhang, Siyun Xiang, Jing Wen, Wen Wang, Ke Guan, Weixiang Wang, Yang Yang, Liuwei Hao, Yongchun Chen

**Affiliations:** ^1^Department of Nutrition, Henan Provincial People’s Hospital and Zhengzhou University People’s Hospital, Zhengzhou, China; ^2^Department of Ultrasound, Henan Provincial People’s Hospital and Zhengzhou University People’s Hospital, Zhengzhou, China; ^3^Department of Reproductive Medicine, The Third Affiliated Hospital of Zhengzhou University, Zhengzhou, China; ^4^Department of Health Management, Henan Provincial People’s Hospital and Zhengzhou University People’s Hospital, Zhengzhou, China

**Keywords:** cardiovascular risk, influencing factors, prevalence, China, CIMT

## Abstract

**Background:**

Carotid intima-media thickness (CIMT) has been shown to be a valuable predictor of cardiovascular diseases (CVDs). The aim of this study was to investigate the influencing factors of CIMT among adults in Central China.

**Methods:**

A total of 2,578 participants who underwent physical examination in Henan Provincial People’s Hospital between January 2018 and July 2018 were enrolled. The respondents were divided into two subgroups according to their CIMT value (CIMT ≥1.0 mm group and CIMT <1.0 mm group). Variables considered were age, gender, total cholesterol, triglycerides, low-density lipoprotein (LDL) and high-density lipoprotein (HDL) triglycerides, fasting blood glucose, and uric acid levels, as well as hypertension, diabetes, body mass index (BMI), waist-to-hip ratio, body fat percentage, and visceral fat area (VFA). Logistic regression analysis was performed to explore the potential factors influencing CIMT.

**Results:**

The proportion of CIMT ≥1.0 mm among the physical examination population was 27.42% (707/2 578). The analysis of the two groups revealed significant differences in age, sex, hypertension, diabetes, total cholesterol, and HDL cholesterol. In the logistic regression analysis, age (*OR*=1.071, 95%*CI*=1.062–1.080), male sex (*OR*=2.012, 95%*CI*=1.251–2.238), hypertension (*OR*=1.337, 95%*CI*=1.031–1.735), diabetes (*OR*=1.535, 95%*CI*=1.045–2.255), HDL cholesterol (*OR*=0.540, 95%*CI*=0.366–0.796), and LDL cholesterol (*OR*=1.336, 95%*CI*=1.154–1.544) were significantly associated with abnormal CIMT.

**Conclusion:**

Early screening should be carried out among men, the older adults, and those with hypertension, diabetes, and dyslipidemia.

## 1 Introduction

Cardiovascular diseases (CVDs) have become a leading cause of morbidity, mortality, and disability in most countries globally (1). According to the data of the 2019 global disease burden report, from 1990 to 2019, the number of patients with CVDs increased from 271 million to 523 million, and the number of deaths increased from 12.1 million to 18.6 million (2). The prevalence of CVDs in China is on the rise. According to the data of China cardiovascular health and disease report 2020, the current number of patients with CVDs is estimated to be 330 million, and there is one patient with CVDs for every five people (3). Therefore, it is essential to reduce the risk of CVDs by exploring preventive methods.

Carotid intima-media thickness (CIMT), measured using noninvasive high-resolution ultrasound techniques, has been shown as a predictor of CVDs that is independent of the routine vascular risk factors (4). In a Japanese cohort study of 4,724 participants aged 59.7 ± 11.9 years, those with Max CIMT ≥1.7 mm had a 1.95-fold higher risk of CVDs than those with Max CIMT <1.7 mm (5). Willeit (6) carried out a meta-analysis on the relationship between CIMT and the risk of CVDs, and systematically reviewed the results of 119 randomized controlled trials, which showed that each 10 μm/year reduction of CIMT progression resulted in a relative risk of CVDs of 0.91. Previous studies have supported the use of CIMT as an alternative marker of CVDs risk, and intervention on the progress of CIMT could effectively reduce the risk of CVDs.

In China, most previous studies on CIMT have focused on its relationship with CVDs (7, 8), few studies have focused on the influencing factors of CIMT, and most of them have studied specific diseases (9). There is no research on the influencing factors of CIMT in Chinese adults who undergo physical examination. Therefore, the aim of this study was to investigate the prevalence of CIMT and explore the potential risk factors for increased CIMT in a physical examination population in central China.

## 2 Materials and methods

### 2.1 Study population

Participants who underwent medical examination in the physical examination center of a tertiary hospital in Henan Province, China, from January 2018 to July 2018 were retrospectively included. If The participants meet the following criteria were included: (1) age ≥18 years old; (2) availability of ultrasound examination of the internal carotid artery; and (3) availability of body fat composition examination results using bioimpedance analysis (BIA). The following participants were excluded: (1) those who had undergone internal carotid plaque surgery; (2) those with a history of administration of lipid-lowering drugs. All the information was obtained from health examination records and inquiries before health examination. Finally, 2,578 participants were included in the study. The study design was approved by the ethics committee of participating institutes. All methods were performed in accordance with the relevant guidelines and regulations by including a statement in the methods section to this effect. All participants had provided written informed consent and their personal information was kept confidential.

### 2.2 Measurements of CIMT

The CIMT was measured using high-resolution B-mode ultrasonography with a 7.5-MHz linear array scan (EPIQ7 ultrasound system, Philips Healthcare, Bothell, WA, USA) by professional radiologists. The bilateral carotid arteries of each participant were examined by longitudinal and transverse scanning with the participant in the supine position during the diastolic phase of the cardiac cycle. CIMT was defined as the distance between the leading edge of the lumen interface and the media-adventitial interface of the far wall. Measurements were obtained on each side at 1–2 cm proximal to the bifurcation of the posterior carotid wall. Each artery was measured three times, and the CIMT value represented the average value of six measurements. An increased CIMT was defined as a value of ≥1.0 mm (10). Thus, participants were divided into two groups: normal CIMT (CIMT<1.0 mm) and abnormal CIMT (CIMT ≥1.0 mm).

### 2.3 Clinical data and biochemical analysis

Venous blood was collected in the morning after 12-h overnight fasting. Measurements were performed using routine laboratory methods for serum parameters such as total cholesterol, triglyceride, low-density lipoprotein (LDL) and high-density lipoprotein (HDL) triglyceride, fasting blood glucose, and uric acid. Diabetes was diagnosed if a participant had a fasting blood glucose level of ≥7.0 mmol/L, a 2-h postprandial blood glucose level of >11.1 mmol/L, or diabetes had been diagnosed before the investigation. Hypertension was diagnosed if systolic blood pressure (SBP) was ≥140 mmHg, if diastolic blood pressure (DBP) was ≥90 mmHg, or if the patient had self-reported hypertension.

### 2.4 Body composition measurement

Body composition was measured by BIA using the InBody 770 (InBody Co., Ltd., Seoul, Korea), which could estimate body mass index (BMI), waist-to-hip ratio, body fat percentage, and visceral fat area (VFA). Waist-to-hip ratio was classified as normal or over-standard with cut off levels for normal classification of 0.9 for males and 0.8 for females.

### 2.5 Statistical analysis

All statistical analyses were performed using IBM statistics (SPSS) V.22. The continuous variables are expressed as means ± standard deviation, and categorical variables are expressed as counts and percentages. Comparisons between the two groups were performed using the Student’s t-test and the Chi-square test. Multivariable logistic regression analysis was used to assess independent relationships for several explanatory variables determined abnormal on CIMT. The odds ratios (OR) and their 95% confidence intervals (95% *CI*) were used to present correlations. *P*<0.05 was considered statistically significant.

## 3 Results

Overall, 2,578 participants were included in this study, including 1,803 (69.94%) males and 775 (30.06%) females. The average age of the participants was 55.36 ± 14.17 years. A total of 404 participants (15.73%) were deemed obese (BMI >27.9 kg/m^2^), and 1,866 (72.38%) participants were in the over-standard group on the basis of waist-to-hip ratio. The average body fat percentage and VFA of the participants was 28.26% ± 7.13% and 94.76 ± 34.01 cm^2^, respectively. Among the participants, 144 (12.53%) had type 2 diabetes mellitus and 422 (16.37%) had hypertension. There were 707 (27.42%) participants with a CIMT greater than or equal to 1.0 mm. **Table 1** shows the basic characteristics of the study population.

**Table 1:** Characteristics of the study population.

Characteristics		Number(%)/Mean(SD)
Gender	Male	1803(69.94)
	Female	775(30.06)
Age(years)		55.36(14.17)
BMI (kg/m^2^)	<24.0	1033(40.23)
	24.0~27.9	1131(44.04)
	>27.9	404(15.73)
Waist-to-hip ratio	Normal	712(27.62)
	Over-standard	1866(72.38)
Type 2 diabetes	Yes	144(5.59)
	No	2434(94.41)
Hypertension	Yes	422(16.37)
	No	2156(83.63)
CIMT (mm)	<1.0	1871(72.58)
	≥1.0	707(27.42)
Total cholesterol (mg/dL)		4.86(0.94)
Triglycerides (mg/dL)		1.85(1.32)
HDL cholesterol (mg/dL)		1.29(0.29)
LDL cholesterol (mg/dL)		2.59(0.70)
Uric acid (μmol/L)		356.36(92.15)
Body fat percentage (%)		28.26(7.13)
VFA(cm^2^)		94.76(33.24)

The proportion of CIMT ≥1.0 mm in males was 32.29%, which was significantly higher than that in females (15.87%), *P*<0.001. The incidence of diabetes and hypertension was higher in the CIMT ≥1.0 mm group than in the CIMT <1.0 mm group (*P*<0.001). Participants tended to be older in the CIMT ≥1.0 mm group compared to the CIMT <1.0 mm group (63.89 ± 13.07 years vs. 50.89 ± 13.09 years, respectively, *P*<0.001). Total cholesterol and HDL cholesterol were also found to be significantly different in the CIMT ≥1.0 mm group than in the control subjects. There were no significant differences in BMI, waist-to-hip ratio, triglycerides, LDL cholesterol, uric acid, body fat percentage, and VFA between the two groups (**Table 2**).

**Table 2:** Comparison of basic information of the two groups.

Characteristics		CIMT(mm)		*t*/*χ^2^*	*P*
		<1.0	≥1.0		
Gender	Male	1219(67.61)	584(32.29)	74.314	<0.001
	Female	652(84.13)	123(15.87)		
Type 2 diabetes	Yes	1806(74.20)	628(25.80)	57.685	<0.001
	No	65(45.14)	79(54.86)		
Hypertension	Yes	1651(76.58)	505(23.42)	105.951	<0.001
	No	220(52.13)	202(47.87)		
BMI (kg/m^2^)	<24.0	743(71.93)	290(28.07)	1.475	0.478
	24.0~27.9	817(72.24)	314(27.76)		
	>27.9	303(75.00)	101(25.00)		
Waist-to-hip ratio	Normal	509(71.49)	203(28.51)	0.584	0.445
	Over-standard	1362(72.99)	504(27.01)		
Age(years)		50.89±13.09	63.89±13.07	22.53	<0.001
Triglycerides (mg/dL)		1.87±1.41	1.79±1.06	1.605	0.109
Total cholesterol (mg/dL)		4.89±0.92	4.79±0.98	2.364	0.018
HDL cholesterol (mg/dL)		1.31±0.29	1.25±0.29	4.236	<0.001
LDL cholesterol (mg/dL)		2.59±0.68	2.58±0.74	0.319	0.750
Uric acid (μmol/L)		354.54±94.17	361.12±86.49	1.613	0.107
VFA(cm^2^)		94.73±33.56	94.85±32.41	0.083	0.934
Body fat percentage (%)		28.26±7.34	28.26±6.53	0.003	0.998

In the multivariable logistical regression analysis, independent abnormal CIMT determinants for the entire group were age (*OR*=1.071, 95%*CI*=1.062–1.080, *P*<0.001), sex (*OR*=1.673, 95%*CI*= 1.251–2.238, *P*=0.001) (ref. female), hypertension (*OR*=1.337, 95%*CI*=1.031–1.735, *P*=0.029), diabetes (*OR*=1.535, 95%*CI*=1.045–2.255, *P*=0.029), HDL cholesterol (*OR*=0.540, 95%*CI*=0.366–0.796, *P*=0.002), and LDL cholesterol (*OR*=1.336, 95%*CI*=1.156–1.544, *P*<0.001). Multivariable regression analysis results are shown in **Table 3**.

**Table 3:** Multiple logistical regression analysis results for variables characterizing the 2,578 subjects and abnormal CIMT.

variables	*β*	S.E.	Wald	*OR* (95%*CI*)	*P*
Age (years)	0.068	0.004	264.097	1.071(1.062-1.080)	<0.001
Gender (ref. female)	0.515	0.149	2.012	1.673(1.251-2.238)	0.001
Hypertension (ref. No)	0.291	0.133	4.790	1.337(1.031-1.735)	0.029
Type 2 diabetes (ref. No)	0.429	0.196	4.768	1.535(1.045-2.255)	0.029
HDL cholesterol (mg/dL)	-0.616	0.198	9.675	0.540(0.366-0.796)	0.002
LDL cholesterol (mg/dL)	0.290	0.074	15.391	1.336(1.156-1.544)	<0.001
Constant	-4.474	0.522	73.563	0.011	<0.001

## 4 Discussion

CVDs have become a worldwide public health problem and a serious threat to human health. Atherosclerosis is the leading cause of CVDs resulting in high rates of mortality and morbidity in the population. Atherosclerosis begins early in life, and it can be latent for a long time before the formation of atherosclerotic plaque (11). Thus, identification of individuals with subclinical atherosclerosis is necessary for early active prevention of CVDs. Previous studies have confirmed that CIMT can be recognized early and should be considered as an important marker of severe atherosclerosis in the future (5, 9). In this study, we found that the prevalence of abnormal CIMT in central China was 27.42%, which was closer to the prevalence reported by Peige et al. in 2018, i.e., 27.22% (12), but significantly higher than the prevalence reported by Yu et al. in 2019, i.e., 3.2% (13). The differences might be partly attributable to the participants’ personal factors (age, sex) and investigation methods (sample selection method, sample size). Thus, it is of great significance to study the CIMT of physical examination population, which can identify the risk factors of CIMT in a time and effectively, and help to prevent the occurrence of CVDs in the early stage.

Logistic regression analysis showed that older age, male sex, hypertension, diabetes, and low HDL cholesterol and high LDL cholesterol levels were independent risk factors for abnormal CIMT. The risk of abnormal CIMT increased 1.071 times for each year of age, which was consistent with the results of Medhioub et al. (14). With increasing age, the metabolic function of the body decreases, the arterial intimal load increases, and the risk of arterial intimal thickening also increases. The risk of abnormal CIMT was 1.673 times higher in males than in females, which may be due to the differences in lifestyle and work stress between males and females. Hypertension and diabetes have been proved to be linked with abnormal CIMT (15–17). Additionally, dyslipidemia was an independent influencing factor for CIMT thickening. Sustained abnormal lipid metabolism would lead to the lipid depositing under the intima, resulting in CIMT thickening, atherosclerosis, and decrease vascular wall compliance (18). Our study indicated that LDL was positively associated with CIMT, which was consistent with previous studies (19–21). However, these studies did not found a relationship between HDL and CIMT. Guo et al. (22) conducted a study on the influencing factors for CIMT in the population of diabetes, and found that LDL was positive related to CIMT, while HDL was negatively corrected with CIMT, which was consistent with the results of our study. Early intervention for high-risk individuals with abnormal CIMT is needed for reducing incidence of CVDs.

It was worth noting that our study did not find a correlation between obesity-related indicators and CIMT, which was inconsistent with previous studies. In 2014, a study on 3,381 healthy people over 40 years of age showed that waist-to-hip ratio and VFA were independently related to increased CIMT; however, no significant correlation was found between BMI and CIMT (23). In addition, a Brazilian survey of 8,449 participants aged between 35 and 74 years of age explored the relationship between CIMT and abdominal obesity, and showed that waist circumference, waist-to-hip ratio and abdominal fat area were significantly correlated with CIMT (24). Yu et al. (13) also analyzed the relationship between abdominal obesity and CIMT and found that there was a correlation between the VFA and increased CIMT. Therefore, more research that explores the relationship between abdominal obesity and CIMT in larger study populations is needed.

Our study had several limitations. First, this study followed a cross-sectional study design, which restricted the predictive effect of the temporality and causality of the observed relationships. Second, the data were collected from participants who voluntarily underwent internal carotid artery ultrasound testing in the health examination center, thus, response bias was unavoidable. Third, other potential risk factors for abnormal CIMT (such as residence, eating habits, daily exercise, smoking, drinking, and other occupational health factors) were not investigated. In the future, community-based multicenter large-sample studies, covering populations from different regions and including more influencing factors, should be conducted to verify the results of this study.

## 5 Conclusion

In conclusion, the results of this study showed that the prevalence of abnormal CIMT was high in a physical examination population in central China. For the older adults people, men, and people with hypertension, diabetes, and dyslipidemia, it is of great importance to carry out early internal carotid artery ultrasound examination. It is also crucial to implement county-level prevention measures to reduce the incidence of CVDs.

## Data availability statement

The raw data supporting the conclusions of this article will be made available by the authors, without undue reservation.

## Ethics statement

The studies involving humans were approved by Henan Provincial People’s Hospital. The studies were conducted in accordance with the local legislation and institutional requirements. The participants provided their written informed consent to participate in this study. Written informed consent was obtained from the individual(s) for the publication of any potentially identifiable images or data included in this article.

## Author contributions

TY: Conceptualization, Project administration, Validation, Writing – original draft. YW: Formal Analysis, Investigation, Writing – review & editing. XZ: Data curation, Writing – original draft. SX: Investigation, Writing – original draft. JW: Investigation, Writing – original draft. WW: Investigation, Writing – original draft. KG: Investigation, Writing – original draft. WXW: Investigation, Writing – original draft. YY: Investigation, Writing – original draft. LH: Investigation, Writing – original draft. YC: Project administration, Resources, Supervision, Writing – review & editing.
